# Treatment of primary vaginal malignant melanoma and review of previous literature: A case report

**DOI:** 10.1097/MD.0000000000036128

**Published:** 2023-12-08

**Authors:** Suning Bai, Qi Wu, Liyun Song, Wenfei Wu

**Affiliations:** a Department of Gynecology, Hebei General Hospital, Shijiazhuang, China.

**Keywords:** chemotherapy, immunotherapy, primary vaginal malignant melanoma, surgical treatment, targeted therapy

## Abstract

**Introduction:**

Primary vaginal malignant melanoma is a rare gynecological malignant tumor with high malignancy and poor prognosis. Because of its insidious incidence, it is generally diagnosed in the late stage, and the 5-year survival rate is only 5% to 25%. Due to the rarity of this disease and the limited number of related cases reported in the literature, there is currently no unified standard for its diagnosis and treatment. Therefore, the treatment of this disease has always been a difficult problem in clinical practice.

**Patient concerns:**

A 56-year-old woman was admitted to our hospital with discomfort in the lower abdomen.

**Diagnosis:**

The final diagnosis of this patient was vaginal malignant melanoma (T4N1M0).

**Interventions:**

The patient underwent extensive hysterectomy, bilateral adnexectomy, pelvic lymph node resection, and total vaginectomy. Following the surgery, the patient received adjuvant chemotherapy.

**Outcomes:**

The patient was followed up regularly. No recurrence or metastasis has been reported to date.

**Conclusion:**

The treatment of primary vaginal malignant melanoma is still dominated by surgery, while radiotherapy and chemotherapy are controversial. Immunotherapy and targeted therapy highlight certain advantages in advanced patients, which still need to be verified by large sample studies, We provide a case of postoperative adjuvant chemotherapy for vaginal malignant melanoma. So far, no signs of disease recurrence have been found. As the price of chemotherapy drugs decreases, it is economically convenient and acceptable for most patients, but its effectiveness needs to be observed in large-scale clinical trials.

## 1. Introduction

Primary vaginal malignant melanoma is a rare gynecological malignant tumor, its etiology is still unclear. Primary vaginal malignant melanoma is insidious, usually diagnosed in late stage, with high malignancy, easy to spread and metastasize in early stage, and poor prognosis. Its treatment is mainly surgical treatment, in addition to immunotherapy, chemotherapy, targeted therapy, radiotherapy, etc. Here, we introduce a 56 years old patient with primary vaginal malignant melanoma and review the literature.

## 2. Case report

**Figure 1.**
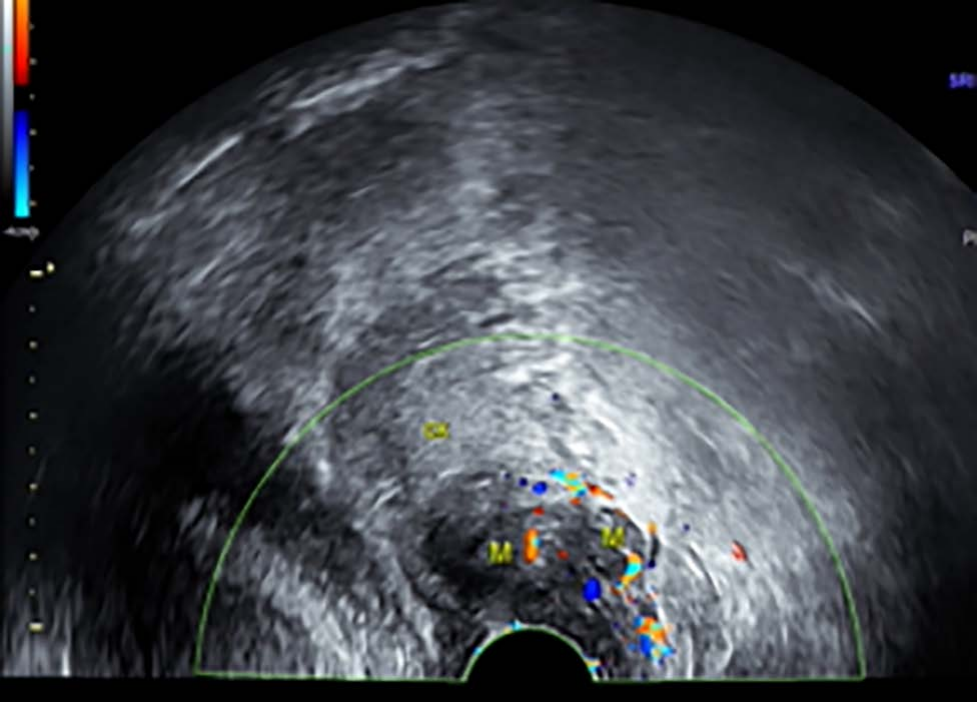
Transvaginal ultrasonography showed low echogenicity in the cervix and low echogenicity in the vagina (excluding cervical fibroids) (a low echogenicity area of approximately 25 × 22 × 20 mm in size and approximately 13 mm in diameter can be seen on the left side of the vagina, closely related to the left anterior wall of the cervix).

A 56-year-old perimenopausal Chinese woman visited our department on January 28, 2023 due to discomfort in the lower abdomen for 5 days, she did not have any abnormal vaginal bleeding or discharge. Her gynecological ultrasound showed that the uterus was of normal size, with a high echogenic mass (approximately 9 × 10 × 6 mm in size) in the uterus, and low echogenicity in the cervix, low echogenicity in the vagina (excluding cervical fibroids; a low echogenicity area of approximately 25 × 22 × 20 mm in size and approximately 13 mm in diameter can be seen on the left side of the vagina, closely related to the left anterior wall of the cervix; Fig. 1). She had menarche at the age of 19, with a normal menstrual period of 5 days, a menstrual cycle of 25–35 days, and her last menstrual period was January 17, 2023. She had no dysmenorrhea and no history of irregular vaginal bleeding. She had 3 children and had 2 abortions. Her BMI is 24.53.

The patient has not undergone routine gynecological examinations before. She had a history of cesarean section, no history of hypertension, diabetes and other chronic diseases, and denied a family history of malignant tumors. After the patient was hospitalized, we conducted a detailed physical examination on her. There were no abnormalities in her heart and lungs, and there was no palpable enlargement of the superficial lymph nodes. There is no tenderness in the abdomen and no palpable mass. During gynecological examination, a lump of approximately 4 × 2 × 1 cm in size can be seen on the left wall of the vagina, which is hard, inactive, and has no tenderness. There is no palpable abnormality in the uterus or double adnexal area. The human papillomavirus and Thin Prep cytology tests of the cervix were both negative. After the patient was admitted to the hospital, all preoperative examinations were completed and there were no abnormalities in the results of her female tumor markers. On January 31, 2023, we performed surgical treatment on the patient. Under hysteroscopy: it was observed that the posterior lip of the cervix and its near outer opening were black and hard in the cervical canal, and no obvious vegetation was found in the cervical canal. The uterine cavity had a regular shape, with a thick endometrium. Multiple vegetation protrusions of different sizes were visible in the uterine cavity, which were white and had a regular shape. The larger one is located on the posterior wall of the uterus, with a diameter of approximately 1.5 cm × 1 cm × 0.8 cm, bilateral fallopian tube openings are clearly visible, and hysteroscopic hysterectomy is performed. The uterine cavity is gently scraped with a small curette for 2 weeks, and approximately 10 g of tissue is removed from the uterine cavity with oval forceps. When placed again under hysteroscopy, all vegetative protrusions are removed, and there is no obvious active bleeding. Bilateral fallopian tube openings are visible. Withdraw hysteroscopic instruments. Disinfect the vagina again, place a catheter, place a vaginal hook, expose the cervix and vagina, and see a protruding lump on the left wall of the vagina, with a size of about 4 cm × 3 cm × 2 cm, hard in texture, the internal part of the tumor can be seen to be black after incision, and the texture is relatively poor and brittle, with unclear boundaries with surrounding tissues. A biopsy of the tumor was performed and sent for frozen pathological examination. Frozen pathological report: (vaginal mass) malignant tumor, considering the origin of the mesenchymal lobe, to be further determined by paraffin section. Explain the condition to the authorized person of the patient: Currently, the frozen pathology report is a malignant tumor, with unclear tumor source and pathological results. Additionally, the posterior lip of the cervix may have the same source of lesion, and if surgery continues, it may not be possible to completely remove the tumor. We suggest that he wait for paraffin pathology, undergo cervical biopsy, and improve pelvic imaging examination. After comprehensive evaluation and preparation, surgical treatment can be performed. The authorized person of the patient understood the condition, And he agreed with the current treatment and requested that only vaginal wall mass biopsy, cervical biopsy, and hysteroscopic hysterectomy were performed for this surgery. Postoperative paraffin pathological report is as follows: 1. (Vaginal tumor) combined with immunohistochemical staining, diagnosis of malignant melanoma. Immunohistochemical staining: Vimentin (+), CKpan (−), S100 (+), HMB45 (+), CD117 (+), Melan A (+), Desmin (−), EMA (−), SMA (−), CD34 (−), Ki-67 (active region approximately 50%+). 2. (Cervical) biopsy tissue: malignant melanoma. 3. (Endometrium tissue) Late proliferative stage endometrium.

**Figure 2.**
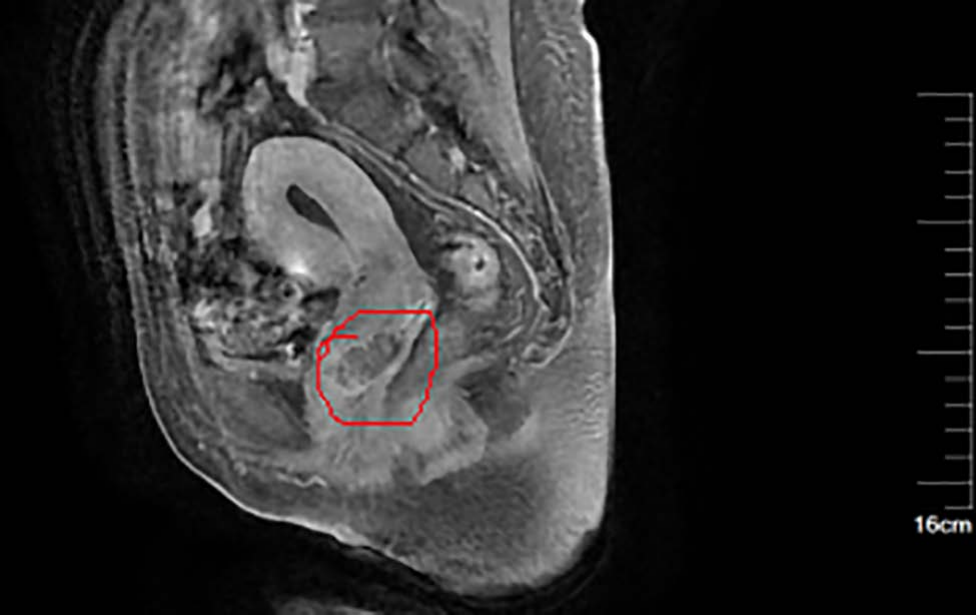
Pelvic MRI showed that there was a high possibility of vaginal malignancy invading the cervix, and abnormal signal shadows in the left adnexal area suggest a high possibility of metastasis.

**Figure 3.**
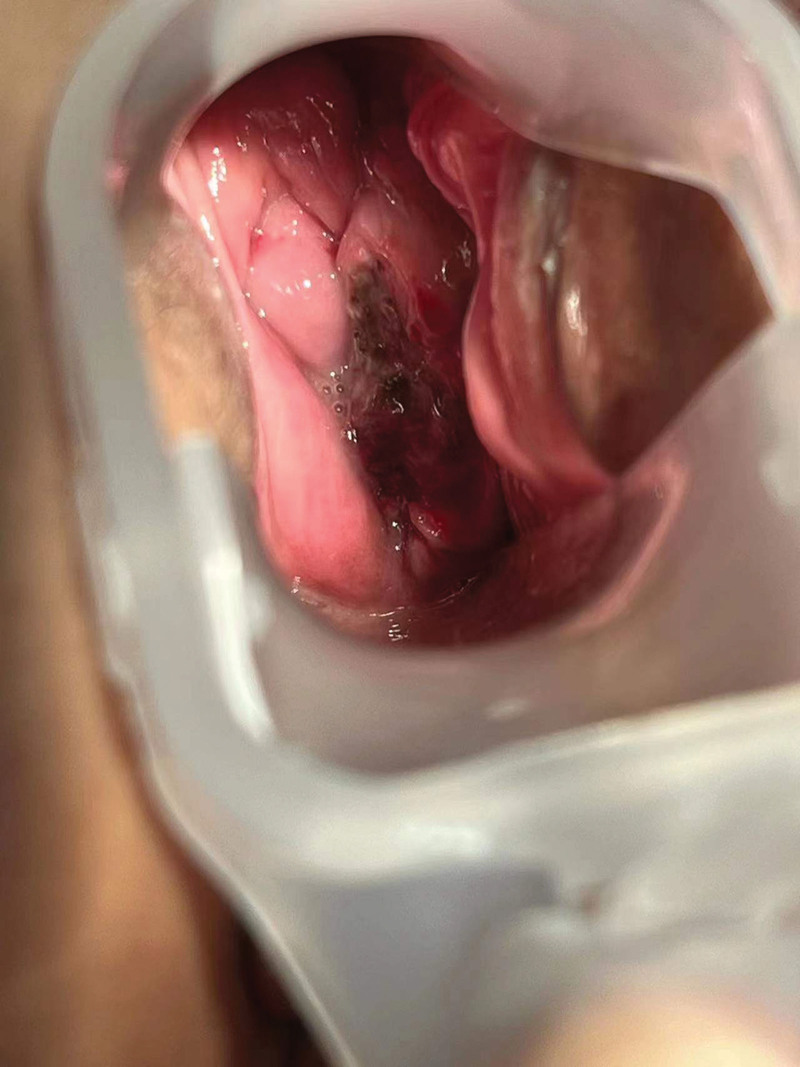
The biopsy wounds of vaginal wall lesions.

**Figure 4.**
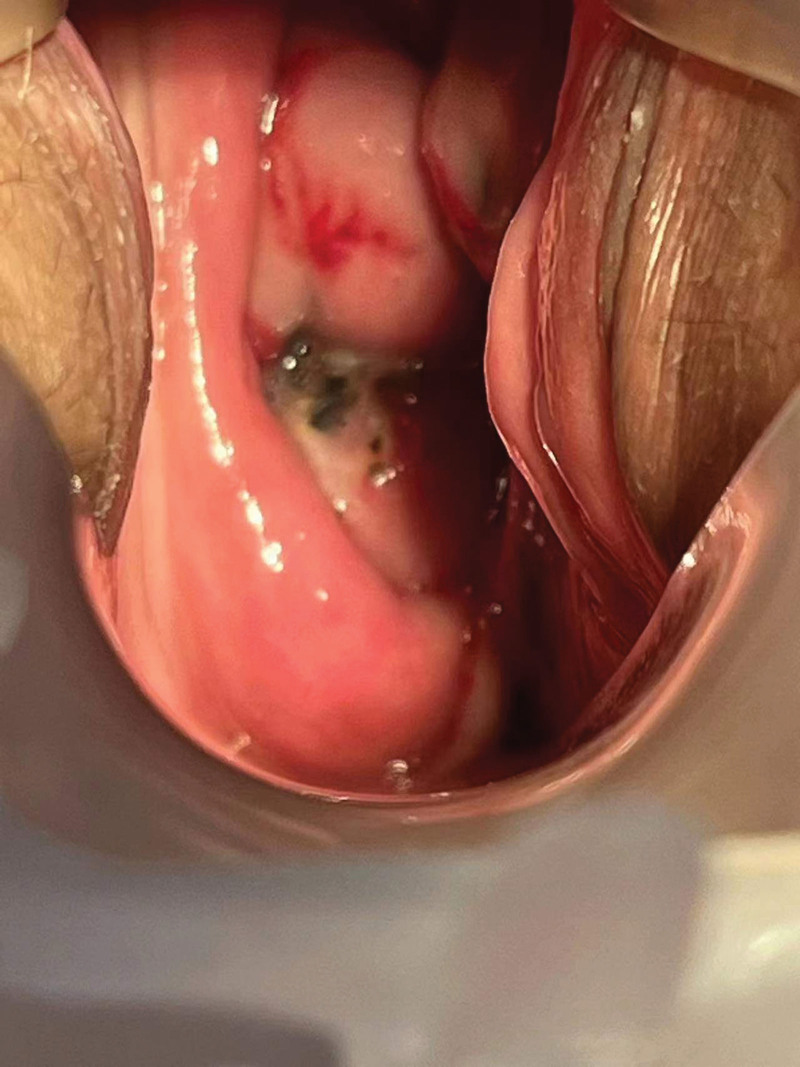
The biopsy wounds of cervical lesions.

We performed abdominal CT, pelvic nuclear magnetic resonance imaging, and enhanced to evaluate this patient’s disease, the abdominal CT did not indicate any detailed abnormalities. The results of pelvic MRI showed that there is a high possibility of vaginal malignancy invading the cervix, and abnormal signal shadows in the left adnexal area suggest a high possibility of metastasis (Fig. 2). We conducted another gynecological examination on the patient to Check the biopsy wounds of vaginal wall lesions (Fig. 3) and cervical lesions (Fig. 4).

**Figure 5.**
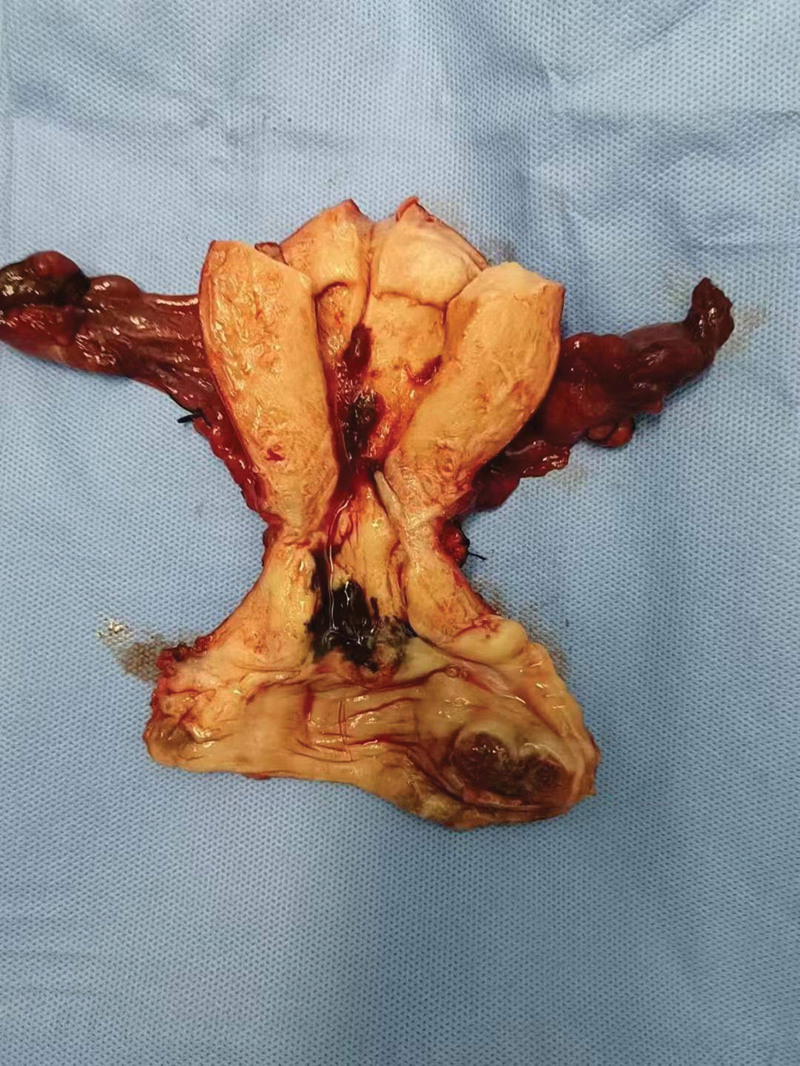
Gross features of the uterus and vaginal wall.

On February 7, 2023, we performed another surgical treatment on the patient, which included extensive abdominal hysterectomy, bilateral adnexectomy, pelvic lymph node resection, left rectal fossa mass resection, severe pelvic adhesions separation, and total vaginal resection. During the operation, it was found that the uterus was slightly larger than normal, the surface was smooth, the appearance of bilateral appendages was normal, and the surface of greater omentum, intestines, liver, and stomach was normal. During the operation, the structure of the right ureter near the bladder was unclear. Urology surgeons were invited to explore the condition of the right ureter on stage, considering that there was no abnormality in the right ureter. During the surgery, a brown and brittle nodule with a diameter of 1.5 cm was observed in the left rectal lateral fossa, indicating the possibility of patient metastasis. After the surgery, we dissected the uterus and vaginal walls, we found that there were no obvious abnormalities in the endometrium, there was a black mass with a diameter of 3 cm on the cervix, and a hard mass with a diameter of 4 cm on the left vaginal wall, black spots on the posterior wall of the vagina (Fig. 5).Postoperative pathological results are as follows: 1. melanoma of the left vaginal wall, nodular type. The local ulcer forms and infiltrates nearly the whole vaginal wall, about 1.3 cm deep, about 20 mitoses/square millimeter. Melanoma can be seen in the cervix. No tumor was found at the broken end of the vaginal wall. Immunohistochemical staining: Vimentin (+), S100 (+), HMB45 (+), CD117 (+), Melan A (+), Ki-67 (approximately 90%+). 2. melanoma can be seen in the left lateral rectal fossa. 3. A total of 21 lymph nodes were removed, and 1 left obturator lymph node was found to have tumor metastasis. The final diagnosis of this patient is vaginal malignant melanoma (T4N1M0). We also suggested that the patient should do gene testing, but no gene mutation was found. We suggest that the patient receive immunotherapy in the future, but due to economic reasons, the patient refused immunotherapy and only received chemotherapy with paclitaxel (albumin-bound; 260 mg/m^2^) combined with carboplatin. So far, she has received 4 courses of chemotherapy, and the patient’s condition is stable, with no tumor recurrence or metastasis found.

## 3. Discussion

Primary vaginal malignant melanoma is a rare malignant tumor of female reproductive tract, accounting for less than 5% of vaginal malignant tumors and less than 1% of all malignant tumors.^[1]^ Its malignancy is high, and the 5-year survival rate is 5% to 25%.^[2]^ It mostly occurs in postmenopausal women aged 60 to 80 years old, and the etiology of the disease is currently unclear. Stimulating chemicals, chronic inflammation, or viral infections are considered to be its pathogenic factors.^[3,4]^ The main clinical manifestations are vaginal bleeding, mostly postmenopausal vaginal bleeding, followed by abnormal vaginal secretions, vaginal lumps, pain, difficulty in sexual intercourse, or itching.^[5]^ The most common site of a lump is the lower one-third of the anterior wall of the female vagina, which can also occur on the posterior and lateral walls of the vagina. It can appear as nodules, polyps, or mushroom shaped ulcers. Ulcers usually form on the surface of the lump, most of which are blue black or gray black, but there are also a few lumps without pigmentation. The patient we reported is a 56 years old perimenopausal woman. The main reason for her diagnosis is abdominal distension. When she was examined by gynecological department, she was found a mass in the middle of the left vaginal wall, and was diagnosed as malignant melanoma of the vagina by focus biopsy.

Pathological section examination is the gold standard for diagnosis of vaginal malignant melanoma, and immunohistochemistry is helpful for diagnosis. Immunohistochemical staining mainly detects S-100 protein, anti-melanoma specific antibody 45 protein, melanoma antigen protein recognized by T cell 1 and SRY-box transcription factor 10 protein. The sensitivity of anti-melanoma specific antibody 45 protein and Melan-A protein is relatively high, so in clinical practice, more than 2 types of proteins are commonly used for joint detection to improve accuracy. The immunohistochemical staining of the patient we reported: S100 (+), HMB45 (+), CD117 (+), Melan A (+), Ki-67 (about 90%+), consistent with the diagnosis of melanoma.

At present, genetic testing is a hot topic in the treatment of various malignant tumors, including malignant melanoma, because genetic testing is not only helpful in the diagnosis of difficult cases, but also helps to guide the application of targeted drugs. At present, some studies have found that mutations that activate SF3B1 and KIT, the deletion of CDKN2A, PTEN, or SPRED1, and the amplification of CDK4, TERT, KIT, MDM2, or CCND1 are common in mucosal melanoma. BRAF and NRAS mutations widely exist in cutaneous melanoma, but are not common in mucinous melanoma.^[3]^ The patient we reported also tested for genes, but unfortunately, no genetic mutations were detected.

Because vaginal malignant melanoma is relatively rare in clinical practice, there is no recommended staging system at present, and its treatment scheme and prognostic factors are not completely clear. At present, it is generally believed that the surgical treatment of vaginal malignant melanoma has obvious survival benefits, so its treatment is still dominated by surgery, adjuvant chemotherapy, radiotherapy, immunotherapy and targeted therapy. Surgery includes local expanded resection and radical resection, with the aim of achieving a negative margin. However, there is no clear definition of the specific distance between the negative margins,^[6]^ and the specific surgical scope is also controversial. Excessive surgical scope may not benefit survival and may also increase postoperative complications. For vaginal malignant melanoma, because of its special location, if the focus is extensive and diffuse, total Vaginectomy, with/without hysterectomy, with/with not double appendage excision is still recommended. Due to the low rate of lymph node metastasis, regional lymph node resection is not routine for patients who do not show positive lymph node results in clinical or impact studies. Radiotherapy is usually used for late stage patients who cannot completely rely on surgery to remove lesions or for patients with postoperative recurrence or metastasis. It is not commonly used for postoperative supplementary radiotherapy and preoperative radiotherapy. Chemotherapy is still controversial in the postoperative adjuvant treatment of vulvar and vaginal melanoma. Some studies have found that it may prolong the OS of patients,^[7]^ and some studies have found that it may prolong PFS, but not OS.^[8]^At present, immunocheckpoint inhibitors have obvious advantages in advanced or metastatic melanoma. At present, Nivolumab, Pamlizumab, or Nivolumab combined with Yipimumab are used as first-line treatment drugs for unresectable or metastatic melanoma.^[9–11]^ ASCO recommends that this scheme is also applicable to mucosal melanoma.^[10,11]^ A study reported a 55 years old patient with vaginal malignant melanoma, After surgery, the primary tumor relapsed with adrenal metastasis. After receiving radiotherapy (RT), anti angiogenic therapy and immunocheckpoint inhibitor, the primary tumor and adrenal metastasis of the patient were smaller than before. The successful case of this treatment suggests that combined treatment can successfully improve the prognosis of the patient and extend the total survival period of the patient. This provides a very useful reference value for the treatment of distant metastatic vaginal melanoma.^[12]^ Another study reported that a 58 years old patient with advanced vaginal malignant melanoma received nivolumab immunotherapy after local lesion resection and had a good prognosis.^[13]^ These findings suggest that for patients with advanced vaginal malignant melanoma, local focus resection combined with postoperative immunotherapy may be a feasible treatment for patients with advanced vaginal malignant melanoma, but this needs to be further confirmed by a large sample of clinical trials. There are also many attempts to target drugs in the treatment of vulvar and vaginal melanoma. Tyrosine kinase inhibitor, such as Imatinib and Nilotinib, have been proved to be useful for cases with genetic C-KIT changes, while larotinib is useful for patients with NTRK gene fusion positive.^[14–16]^ Our patient finally received transabdominal extensive Hysterectomy, bilateral adnexectomy, pelvic lymph node resection, left lateral rectal fossa tumor resection, pelvic severe adhesion separation and total Vaginectomy. Although the scope of surgery is large, fortunately, the patient did not have postoperative complications. Finally, pathology confirmed that there was a tumor metastasis in one left obturator lymph node of the patient, and the focus of melanoma can also be seen in the rectal fossa. We suggest that the patient apply immunotherapy after surgery, but due to the current high cost of immunosuppressive drugs for immune checkpoint inhibitors, the patient’s economic conditions are relatively difficult and they cannot afford the cost of immunotherapy. Therefore, we proposed chemotherapy to her, and the patient received chemotherapy. Although the effect of chemotherapy in the treatment of vaginal melanoma is controversial, chemotherapy drugs are cheap and acceptable for most patients.

## 4. Conclusion

Primary vaginal malignant melanoma is a rare malignant tumor of the female reproductive tract. At present, its pathogenesis is not clear, and the staging system and treatment have not yet formed a consistent standard and guide. So far, its treatment is still dominated by surgery, while radiotherapy and chemotherapy are controversial. Immunotherapy and Targeted therapy highlight certain advantages in advanced patients, which still need to be verified by large sample studies, Moreover, due to its current high price, there are certain limitations to its clinical application. We provide a case of postoperative adjuvant chemotherapy for vaginal malignant melanoma. So far, no signs of disease recurrence have been found. As the price of chemotherapy drugs decreases, it is economically convenient and acceptable for most patients, but its effectiveness needs to be observed in large-scale clinical trials. In short, there are many aspects of vaginal melanoma that are worth exploring by clinicians. We should gradually accumulate relevant cases in clinical work, and strive to find effective treatment methods as soon as possible to benefit patients with this disease.

### 4.1. Limitations of the study

The follow-up time of this case is relatively short. Although the patient’s condition is stable and there is no disease progression during the follow-up time. To evaluate the effectiveness of the current treatment method, it is necessary to conduct lifelong follow-up to understand the patient’s disease development, accurately record the patient’s progression free survival time and total survival time, and such data is persuasive. In addition, this study is only a case study, and the standard and effective treatment methods for vaginal malignant melanoma need to be further explored, and large-scale clinical trials need to be conducted for research.

## Author contributions

**Conceptualization:** Suning Bai.

**Formal analysis:** Suning Bai.

**Investigation:** Suning Bai.

**Resources:** Suning Bai.

**Software:** Suning Bai, Qi Wu, Liyun Song, Wenfei Wu.

**Supervision:** Suning Bai.

**Visualization:** Suning Bai.

**Writing – original draft:** Suning Bai.

**Writing – review & editing:** Suning Bai, Qi Wu, Liyun Song.
